# An Adaptive LoRaWAN MAC Protocol for Event Detection Applications

**DOI:** 10.3390/s22093538

**Published:** 2022-05-06

**Authors:** Athanasios Tsakmakis, Anastasios Valkanis, Georgia Beletsioti, Konstantinos Kantelis, Petros Nicopolitidis, Georgios Papadimitriou

**Affiliations:** Department of Informatics, Aristotle University of Thessaloniki, 54124 Thessaloniki, Greece; atsakmaki@csd.auth.gr (A.T.); valkanae@csd.auth.gr (A.V.); gmpelets@csd.auth.gr (G.B.); kkantelis@csd.auth.gr (K.K.); gp@csd.auth.gr (G.P.)

**Keywords:** Internet of Things (IoT), LoRaWAN, learning automata, delay

## Abstract

In recent years, the Internet of Things (IoT) is growing rapidly and gaining ground in a variety of fields. Such fields are environmental disasters, such as forest fires, that are becoming more common because of the environmental crisis and there is a need to properly manage them. Therefore, utilizing IoT for event detection and monitoring is an effective solution. A technique for monitoring such events over a large area is proposed in this research. This work makes use of the Long-Range Wide Area Network (LoRaWAN) protocol, which is capable to connect low-power devices distributed on large geographical areas. A learning-automata-based hybrid MAC model is suggested to reduce the transmission delay, when a small part of the network produces event packets stemming from an event occurrence that is related to environmental monitoring applications, such as events related to forest fires. The proposed hybrid MAC is evaluated via simulation, which indicates that it achieves significantly higher performance in terms of packet delay, when compared to traditional LoRaWAN schemes.

## 1. Introduction

Since the emergence of IoT networks, there has been extensive research and development for the most suitable technology to be used for communication of IoT. Among them, Low-Power Wide Area Networks (LPWANs) allow communication between a variety of electronic devices and sensors, while their lightweight nature has already led to several implementations on the market [1]. The biggest application area seems to be smart cities, with applications including smart parking, air pollution monitoring, etc. [2].

A well-known LPWAN technology is LoRaWAN. It is an open standard built on the top of the proprietary LoRa [3] physical layer that fits the requirements of IoT networks, while also provides low energy consumption, long communication range and minimal cost of deployment. Additionally, the fact that everyone can use the radio frequencies in a LoRaWAN Wireless Sensor Network (WSN) without paying an extra fee for transmission rights, has sparked the global interest in this technology [4].

MAC protocols are usually focused on specific network scenarios. However, not all protocols are suitable for all cases. For example, TDMA-based protocols are appropriate when there is higher medium usage, since they are not sensitive to interference from concurrent transmissions, as are contention-based protocols such as ALOHA and CSMA. More specifically, in TDMA, nodes transmit at specific pre-defined times. As a result, it is more effective for applications sensitive to packet loss. Despite that, in TDMA all nodes must follow the same transmission schedule. Thus, clock synchronization is required, which is a factor that affects the duration of the transmission slots [5].

On the other hand, ALOHA-based protocols have better performance in scenarios of low to medium load [6]. In ALOHA implementations, nodes try to transmit whenever they have new data, rendering the protocol suitable for elastic traffic. When few nodes attempt to access the medium, high throughput is achieved. However, when more nodes in high load situations attempt to access simultaneously, undesired collisions occur, and the packet delay is increased due to retransmissions. Additionally, some nodes may be blocked, as their transmission collides repeatedly [7]. As a result, considering the conditions of the network, an automatic and adaptive switch between TDMA and ALOHA according to the network load is an efficient solution. Furthermore, Slotted ALOHA constitutes a MAC protocol that has been extensively proposed for LoRaWAN networks [8,9].

In the light of the aforementioned remarks, this paper focuses on the detection and monitoring events such as forest fires in LoRaWAN environments, by using machine learning methods. The functionality of artificial intelligence and machine learning is widely deployed in all sectors, such as agriculture, health and industry. A learning-automata-based hybrid model is proposed to facilitate traffic in the presence of events, in order to facilitate the transmission of event packets. The main goal of the learning automaton is to reduce the transmission delay of event packets, containing critical information, as the corresponding sensors detected suspicious and abnormal measurements such as those regarding a new or ongoing fire [10]. A maximum response time limit for fire monitoring environments is defined at approximately 10 min [11,12]. The suggested hybrid model selects the most appropriate MAC protocol, among a set of available MAC protocols, depending on the network traffic load. Finally, the learning automaton uses a feedback mechanism to constantly adapt to changes in the environment. The contribution of the article is reflected in the reduction of delay in cases of monitoring applications, where event traffic is generated, for LoRa networks supporting energy saving (such as the approach against which the proposed work is compared).

The structure of this paper continues as follows. Section 2 presents the related works on this field, while Section 3 describes the LoRaWAN protocol and its architecture. In Section 4, the learning-automata mechanism is defined. Section 5 analyzes the network environment and the proposed hybrid model. In Section 6, the simulation results are presented. Finally, constructive conclusions are reported in Section 7.

## 2. Related Work

This section presents several related works which involve mechanisms or hybrid systems for the selection of existing MAC protocols in distinct types of networks. This review is not focused on the MAC protocols themselves, instead it concentrates on the selection method of them.

Asudeh et al. [13] proposed a selection framework to choose the appropriate protocol that satisfies the requirements for a given context, defined by a set of input parameters, in a WSN. Three categories of protocols are clearly marked, and it is assumed that protocols in the same category have similar performance characteristics. The authors defined a combined performance function that relates different metrics, such as delay and energy consumption, into a single scalar measure by scaling appropriately each metric. The aim of this performance function is to quantify the performance of each protocol, and after that to choose the most appropriate one, regarding particular context and application requirements.

Jefferson et al. [14] developed a flexible platform to change the active MAC protocol in the network. Nevertheless, the MAC protocol selection algorithm is limited. Its logic depends on the knowledge of an external agent, to accurately tune the inference engine. A disadvantage of the system is the fact that the addition of new protocols requires changes, as it increases the complexity. Furthermore, the model is static, meaning that it does not learn from the environment and thus it does not adapt to changes over time.

Gomes et al. [15] deployed a decision algorithm for unknown medium dynamics. The authors use reinforcement learning techniques to develop a Self-Organizing MAC (SOMAC) sublayer to switch MAC protocols in structured networks, which adapts to network variations. Finally, SOMAC’s decision model can be extended to accept new MAC protocols, without changing the reinforcement learning engine.

Jooris et al. [16] developed a hardware framework, referred to as TAISC (Time-Annotated Instruction Set Computer), that aims to simplify the development of new protocols for sensor nodes. It consists of a cross-platform MAC protocol compiler and an execution engine. This design allows the upgrade as well as the implementation of multiple MAC protocols. These protocols can be switched depending on the network changes or applications needs.

Hsieh et al. [17] proposed a MAC protocol for WSN, to optimize both the energy consumption and the packet latency. The authors proposed a crossing hybrid MAC scheme that dynamically switches the communication for MAC protocol between TDMA and CSMA, based on the routing information of Ad hoc On-Demand Distance Vector (AODV). To minimize the energy consumption, nodes that are not candidates for data transmission, were alternated to schedule their data using the TDMA method. The main disadvantage of this approach is that it is difficult to generalize the solution to other types of networks.

In [18], Ye et al. created an adaptive MAC solution for a fully connected mobile ad hoc network (MANET). The authors established a mathematical relationship between the MAC performance metrics, such as throughput and delay, and the network traffic load indicator, such as the total number of nodes. Two MAC protocols are used: DCF and D-TDMA. Each protocol is associated with a mathematical model. After that, the switching point is derived. Any new MAC protocol necessitates the creation of a new mathematical model, which, in turn, necessitates the network administrator’s technical skills. Therefore, this method lacks scalability for introducing new protocols.

Qiao et al. [19] determined an adaptive MAC protocol selection scheme (SMAC), which is based on machine learning techniques for cognitive radio networks. The available protocols consist of TDMA and CSMA. These protocols are selected by a Sequential Minimal Optimization (SMO) classifier. According to the network loads, SMAC combines the advantages of competitive and non-competitive protocols, and leads network nodes in selecting the MAC protocol that is the most suitable to the current network situation. In fact, SMAC uses supervised machine learning, which necessitates the use of a labeled training set. The authors developed this set via network simulation in which they compared the performance of each protocol under the identical network conditions. This labeling process is difficult to replicate in real-world wireless networks because it depends on external agents that limit the experiment repeatability. Furthermore, because of the non-stationary nature of wireless networks, SMAC is limited to the knowledge gathered from the training set.

Farhad et al. [20] proposed Hybrid Adaptive Data Rate (HADR) for IoT that uses LoRa’s ADR mechanism to improve the performance in cases where there are both fixed and moving users, using the same MAC protocol. HADR constitutes a hybrid model in terms of parameter selection. This approach led to increased packet success rate compared to other approaches, such as Adaptive Data Rate (ADR) and blind Adaptive Data Rate (BADR).

Related works have mentioned that hybrid MAC selection schemes exist, but they do not correspond to LoRaWAN environments. Additionally, there are adaptive schemes for LoRaWAN (HADR) which are not hybrid MAC selection. This work is focused on the implementation of such a hybrid MAC selection scheme, as in an event monitoring application typically data packet measurements are produced at relatively large time intervals. They also need to avoid collisions so as to preserve energy. However, when an event occurs, nodes will want to notify immediately with a random-access method; thus, TDMA is not well suited. The hybrid model is needed to utilize the network information to switch to the best-suited MAC protocol, thus adapting to changing conditions stemming from the transmission (or not) of event packets.

## 3. LoRaWAN Network Architecture

The star network topology is used by the majority of LPWANs. Its nature is determined by a single central node that serves as gateway and connects network end devices. The mesh topology, on the other hand, is made up of individual nodes that communicate with each other to extend the range of communication.

Long Range (LoRa) is a long-range low-power wireless technology platform that employs unlicensed radio spectrum in the Industrial, Scientific and Medical (ISM) radio band to send small packets of data from a low-power transmitter to a receiver. Because of these characteristics, LoRa is classified as a LPWAN. LoRa intends to eliminate repeaters, minimize device costs, extend device battery life and support a large number of devices. Additionally, it makes use of the star topology.

LoRa employs chirp-spread-spectrum (CSS) modulation [21], a spread-spectrum technology that encodes data using linear frequency modulation chirp pulses with a large bandwidth. A chirp pulse is a sinusoidal signal with an increasing or decreasing frequency. A symbol is determined by a chirp. LoRa is the first low-cost infrastructure implementation to be commercialized utilizing CSS. Because of its ability to tolerate interference, CSS has been utilized in long-range communications by military and space agencies [22]. For a certain amount of time, called symbol time (*T_s_*), the chirps change frequency. Three settings are configurable in LoRa. Spreading factor (*SF*) is one of them, and it defines the number of bits that can be encoded in each LoRa symbol. Thus, a symbol can have 2^SF^ values, which are referred to as chirps. *SF* has a range of integer values ranging from 7 to 12 [23]. Another parameter is bandwidth (*BW*), with 125 kHz being the most often used *BW* value in LoRa. Additionally, 250 kHz and 500 kHz bandwidths are provided. The coding rate (*CR*) is the proportion of non-redundant bits for forward error correction (*FEC*) and is the third parameter. By adjusting all these variables, some end-to-end communication features, such as data rate, transmission range and error-correction capabilities, can be adjusted [24]. The symbol rate *Rs* is defined as R_s_ (symbol/s) = BW/2^SF^. Furthermore, coding rate *CR* is calculated according to CR = 4/(4 + *n*), where *n* = 1, 2, 3, 4. The message transmission time, or Time-on-Air (ToA), and the power consumption in the device transmitter both increase as the *SF* increases. However, for a higher *SF* value, the coverage range will be larger because to the increased robustness against noise [25].

Signals received with various *SF*s are mainly considered entirely orthogonal, when using different channels. This means that a LoRaWAN gateway can simultaneously receive several transmissions on various *SF*s and apply this mechanism to each channel [26]. The end node characteristics (*SF* and transmit power) can be configured dependent on the gateway distance, and this feature allows networks with numerous gateways to be run.

At the physical layer, a LoRa packet contains a preamble (usually eight symbols in length), a header, the payload and a cyclic redundancy check (CRC) field. The payload can be 51 to 256 bytes in size, depending on the *SF* and CRC verification codes [23].

Τhe medium access control layer (MAC) and the application layer are defined by LoRaWAN. A LoRaWAN network’s conventional architecture is based on the star network topology, and more specifically, stars-of-stars network, in which a gateway relays communications between an end device and a central network server [27]. The network server routes each device’s data to an application server. The advantages of employing a star topology include preserving battery life and reducing network complexity, as well as the fact that the nodes do not have to act as relays to disseminate or forward data from other nodes because each node only receives its own data.

Even though communication between the end nodes is not supported in LoRaWAN, it is possible to combine it with other specifications that do. All communications between the devices are bidirectional in general [28].

**Figure 1:**
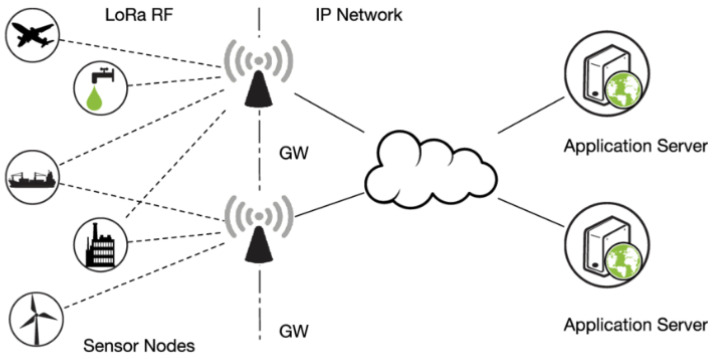
LoRaWAN architecture.

The architecture of LoRaWAN is depicted in Figure 1, which can be separated into front-end and back-end components [29,30]. The front end is composed of gateways and end nodes, meanwhile the back end is made up of network servers that are in charge of security, storing received data, filtering duplicate packets and scheduling acknowledgements through the gateway [31].

**Figure 2:**
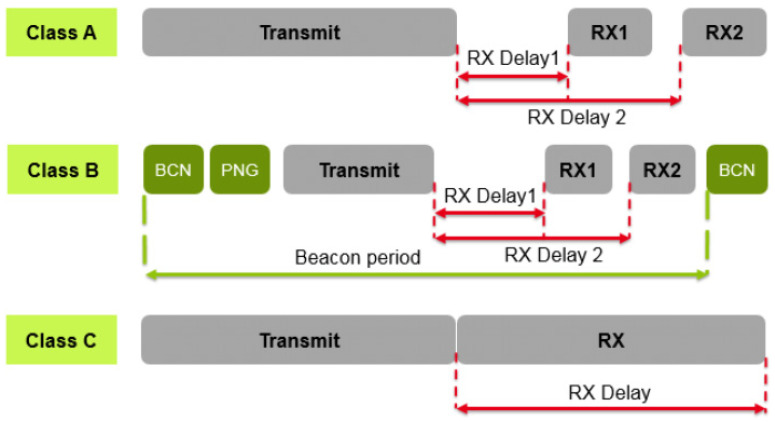
Uplink and downlink methodology in Class A, B and C.

Class A (the default), Class B and Class C are the three operating modes for LoRaWAN end-node devices, depending on the communication instrument used. The end-node device can connect to a wireless network in one of three modes. Class A prioritizes upward traffic between the end-node and the network server, enabling bidirectional communication. As demonstrated in Figure 2, the end-node schedules all transmissions, whereas the server can only send in one of two receive windows that open following a prior uplink transmission. As a result, any packets that the application level needs to send will have to wait until the next receive window opens. Optional features in Class B and Class C increase traffic flow from the network server to the end-node device [32].

Duty cycle, a metric for reducing packet collisions, is a critical LoRaWAN constraint. The maximum percentage of time an end device can occupy a channel is referred to as the duty cycle. In Europe, the LoRa standard is based on a 1% duty cycle. This indicates that just 1% of the time, both end devices and gateways can transmit data. If the ToA is 500 ms, for example, a message can be transmitted again in 99 × 500 = 49.5 s. As a result, a duty cycle of 100 percent indicates that the device can transmit at any moment. A duty cycle of 0%, on the other hand, assumes that the transmitter is always turned off [28].

Wake-up radios (WuR) are a new technology that can continuously monitor the wireless channel while using orders of magnitude less energy than traditional radio hardware used in WSNs. To make use of this technology, sensor nodes are equipped with wake-up receivers and placed in low-power states, waiting for a remote trigger signal.

When a legitimate wake-up beacon is spotted, the main node is woken up and can transmit data “instantaneously”, reducing latency. As a result, the system can be triggered on demand, allowing for “pure” asynchronous communication. The key advantages of this technology are the extended battery lifetime and on-demand downlink communication, both of which are attributable to energy saving [33].

## 4. Learning-Automata Mechanism

The learning-automata theory is based on the concept of an “automaton”. An automaton is a self-operating machine or a mechanism that responds to a set of instructions in a certain way, in order to achieve a specific goal. The automaton either follows a set of pre-determined rules or adjusts to the dynamics of the environment in which it operates. The act of collecting knowledge and adjusting one’s behavior based on that understanding is referred to as “learning”. The automata try to learn the optimum action from a set of actions provided by their random stationary or non-stationary environment [34].

Through repeated interactions with a random environment, a learning automaton [35] improves its performance by learning how to select the best action from a finite set of possible actions. The action is selected at random from a probability distribution over the action set, and the given action is used as the input to the random environment at each instant. The environment responds with a reinforcement signal in response to the token activity. A vector *p*(*n*) = {*p*_1_(*n*), *p*_2_(*n*), *…*, *p_m_*(*n*)} is used by the learning automaton, in order to store the probability distribution for selecting each action (*α*_1_, *α*_2_, *…*, *α_m_*) at cycle *n*. Obviously $\overset{m}{\underset{i=1}{\sum }}p_{i}\left(n\right)=1$. The environmental response *β*(*n*) received, based on the action *ai* selected at cycle *n*, is used to update the action probability vector. This is accomplished using a reinforcement scheme. The automaton determines the next action based on the updated probability distribution vector *p*(*n* + 1) when the updating phase is completed. A learning automaton’s goal is to choose the best action from the action set, so that the average penalty received from the environment is as low as possible [36,37]. The following is the reinforcement scheme that is used in general (1):

(1) $$\begin{array}{l} p_{i}\left(n+1\right)=p_{i}\left(n\right)-\left(1-\beta \left(n\right)\right)*g_{i}\left(p\left(n\right)\right)+\beta \left(n\right)*h_{i}\left(p\left(n\right)\right),\;if\;a\left(n\right)\neq a_{i}\\ p_{i}\left(n+1\right)=p_{i}\left(n\right)+\beta \left(n\right)*\underset{j\neq i}{\sum }\left[g_{j}\left(p\left(n\right)\right)\right]-\beta \left(n\right)*\underset{j\neq i}{\sum }\left[h_{j}\left(p\left(n\right)\right)\right],if\;a\left(n\right)=a_{i} \end{array}$$

The functions *g_i_* and *h_i_* correspond to reward and penalty for the action *a_i_*, respectively, and *β*(*n*) is a measure of the environmental response, normalized in [0, 1]. The lower the value of *β*(*n*), the more favorable the response.

There are three categories of environments based on the type of the reinforcement signal *β*(*n*). These categories are P-model, Q-model and S-model. P-model environments are those in which the reinforcement signal can only take two binary values: 0 and 1. A different type of environment permits the reinforcement signal to take a finite number of the values in the interval [0, 1]. This type of environment is known as Q-model environment. Finally, the reinforcement signal in S-model environments can be any real value in the [0, 1] range [38].

**Figure 3:**
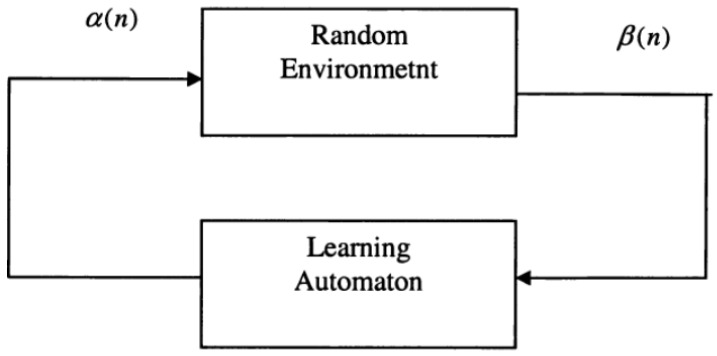
The relationship between the learning automaton and its random environment.

The relationship between a learning automaton and its random environment is depicted in Figure 3. The learning automaton chooses an action *α*(*n*), applies it to the environment, the random environment analyzes the chosen action and emits a response (reinforcement signal *β*(*n*)); finally, the automaton adjusts its state based on the received response.

## 5. The Proposed Hybrid Model

As mentioned before, several systems and mechanisms have been implemented that are related to hybrid MAC protocol selection schemes. However, none have been developed for LoRaWAN networks. This work focuses on the development of a hybrid MAC protocol selection system for LoRaWAN networks, based on machine learning and learning automata, in order to facilitate environmental monitoring applications that generate event packets requiring decreased delays for the reception. Relative applications comprise forest monitoring and fire detection.

### 5.1. The MAC Protocol Selection Algorithm

The proposed method suggests that the server uses a learning automaton, whose probability distribution vector contains the server’s estimate *p_i_* of selecting a MAC protocol. More specifically, the learning automaton periodically selects among a set of available MAC protocols, according to the network’s demands. Therefore, each MAC protocol constitutes an action. The set of available MAC protocols consists of TDMA and Slotted ALOHA, as the latter one has been extensively proposed for LoRaWAN [8,9], and in order to better withstand collisions compared to Pure ALOHA. TDMA is preferred in cases where there is a high network traffic load, to avoid collisions and thus delay increases. On the other hand, Slotted ALOHA is preferred in cases where there is a low network traffic load, to reduce packet delay. The network traffic load is determined by the percentage of the network nodes that need to transmit event packets as fast as possible. Event packets include crucial information, such as increased temperature, smoke detection, sudden change in humidity etc., in the case of a forest fire.

The selection of TDMA leads to the following operation. First, a data request is sent to the cluster head (CH) by the server, which lasts as long as the ToA, and depends on factors such as the used SF, as shown in Table 1. After that, the CH sends a wake-up beacon to the end devices, taking advantage of the short-range capability of the wake-up receivers. Finally, the end devices respond to the request by transmitting their data to the server, regardless of whether the packets are considered as event packet or not, after which they go back to the deep sleep listening state until the next wake-up event is detected. The server transmits an ACK, in order to verify the successful transmission, according to the used SET in each case. The *SF* for an ACK transmission is equal to the *SF* described in Table 1, for each SET, respectively, irrespective of the receive window. For example, in case the end device did not receive an ACK with SET 3 in the first receive window, then the *SF* for ACK transmission in the second receive window is equal to 7. All the end devices are synchronized and each one occupies a specific time slot, to transmit its data, according to its node id. One cluster head is used in total, regardless of the number of end devices.

The selection of Slotted ALOHA instead determines the following operation. Each end device that generated an event packet attempts to transmit it, according to Slotted ALOHA. Therefore, all nodes compete for the channel. In case two or more nodes attempt to transmit at the beginning of the same time slot, a collision occurs, and the collided packets are scheduled for retransmission, based on a backoff algorithm. As a result, frequency collisions are considered, as only one channel is used for uplink transmissions. The rest of the channels are used by other clusters of the system, in order to provide coverage over a large area. In addition, power capturing is not considered, resulting in presenting the worst possible results. So, the end device waits for a random amount of time before retransmitting the data. The only case of successful transmission occurs if the channel is used by a single node. This operation is started by receiving a command by the server, to the CH, which in turn, sends a wake-up beacon to the end-devices, similarly to TDMA. Therefore, in this way, the end devices are informed about how they should transmit. Finally, an ACK is transmitted by the server in case of a successful transmission, similarly to TDMA.

After selecting an action, the learning automaton adapts based on the feedback received from the environment, called environmental response *β*(*n*). When Slotted ALOHA is selected, the environmental response is defined as the percentage of the network nodes that successfully transmitted an event packet in the previous cycle. This happens because the selection of Slotted ALOHA is associated with low network traffic load; therefore, this action only requires a small set of the total number of nodes to transmit event packets, in order to increase the probability of its selection. In other words, Slotted ALOHA requires a few nodes to transmit event packets to be re-selected, and since mathematically this means that *β*(*n*) must be close to zero (as will be explained below), it appears that this environmental response is used. For example, assuming 10 nodes in total, it is recorded how many of these 10 transmitted an event packet. In case only 1 node transmitted (low traffic load, so re-selection of Slotted ALOHA is expected), then *β*(*n*) equals to 0.1, which mathematically leads to the increase in the Slotted ALOHA selection probability, which is what is required as the network traffic load is low. 

On the contrary, when TDMA is selected, the environmental response is defined as the percentage of the network nodes that did not generate event packets in the previous cycle. Therefore, the environmental response correlates with the percentage of the network nodes that either did not transmit at all or transmitted regular packets. The collision-less TDMA is preferred in high network traffic load, and therefore this action requires many nodes to transmit event packets. So, from this point of view, only a few nodes must remain inactive (not to transmit event packets). Considering that *β*(*n*) must be close to zero, in order for this action to be favorable, this environmental response is preferred. For example, assuming 10 nodes, 8 of which transmitted an event packet (high traffic load, so re-selection of TDMA is expected), *β*(*n*) equals to 0.2, as only two nodes remained inactive. As a result, mathematically, the probability of re-selecting TDMA increases, which is desirable as the network traffic load is high.

### 5.2. The Probability Updating Scheme

The probability updating scheme of an S-model linear reward–penalty (*SL_R-P_*) learning automaton is used. When the environment provides a favorable response (low *β*(*n*)) for selecting MAC protocol *i*, the probability estimation of this protocol is increased. On the other hand, when the environment provides an unfavorable response, the probability estimation of MAC protocol *i* is decreased. Clearly, an unfavorable response is received when the selected MAC protocol is not suited for the network’s traffic load. Following the selection of MAC protocol *i* (assuming it is the server’s nth selection), the probability updating scheme described below is employed. On the contrary, the MAC protocol that was not selected is marked as *j*.

(2) $$\begin{array}{c} p_{i}\left(n+1\right)=p_{i}\left(n\right)+L*\left(p_{j}\left(n\right)-a\right)*\left(1-2*\beta \left(n\right)\right)\\ p_{j}\left(n+1\right)=p_{j}\left(n\right)-L*\left(p_{j}\left(n\right)-a\right)*\left(1-2*\beta \left(n\right)\right) \end{array}$$

**Figure 4:**
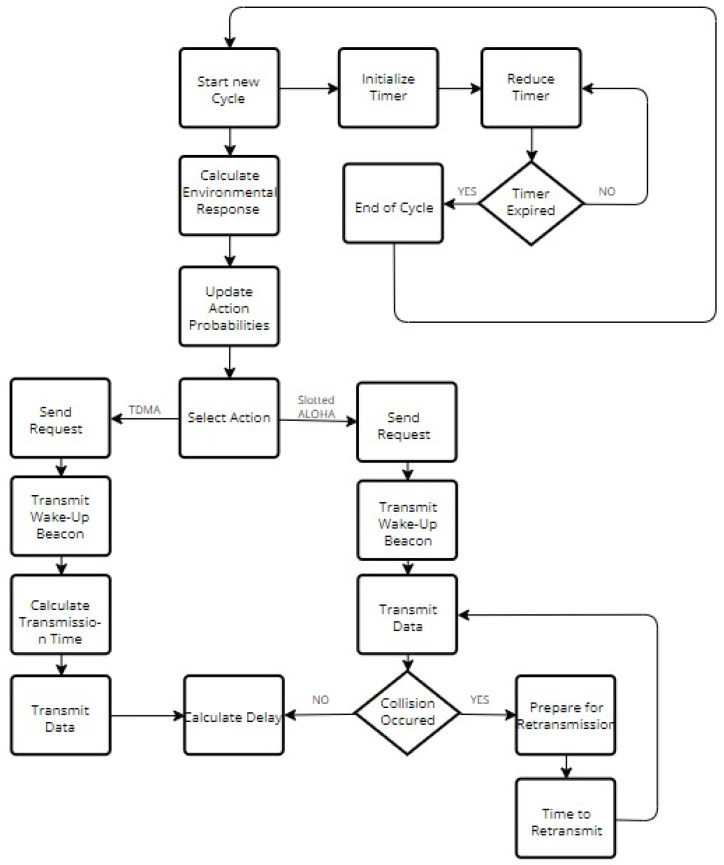
Algorithm flow chart.

Equation (2) is derived from (1) by setting $g_{i}\left(p\left(n\right)\right)=L*\left(p_{i}\left(n\right)-\alpha \right)=\;h_{i}\left(p\left(n\right)\right)$, where *L* is a parameter that regulates the automata convergence speed. The technique for selecting a value of *L* reflects the trade-off between speed and accuracy. When *L* is relatively high, the automaton adapts quickly to network state changes. The automaton’s accuracy, however, is low in this scenario. A comparatively low value of L parameter, on the other hand, results in high accuracy and a slow adaptation rate. Because there is a trade-off between speed and accuracy, the value of the step size parameter *L* must be carefully chosen, in order to produce a speed vs accuracy tradeoff that maximizes network performance. It holds that *L*, *α* ∈ (0, 1) and *p_i_*(*n*) ∈ (α, 1), ∀ i ∈ [1, M], where M is the number of server’s available MAC protocols. The purpose of parameter *α* is to keep the probabilities from approaching zero, thereby increasing the automaton’s adaptivity. Finally, the normalized environmental response, after the server’s nth selection, is illustrated by *β*(*n*). Figure 4 summarizes the flow chart of the algorithm. In addition, the probability updating scheme (Equation set (2)) has a linear complexity to the number of actions, thus the number of employed MAC protocols in this case. As a result, a drawback of the proposed system is the case where several MAC protocols are employed. However, as there are only two protocols, the computation cost is indeed very low. In order to implement the proposed method in a LoRaWAN testbed, a logic module on the Network Server that implements the proposed hybrid MAC selection approach is needed, as the approach of [39,40] has already been implemented in a testbed.

Furthermore, the end devices do not actually connect to the gateway. Rather, they broadcast uplink packets and the gateway forwards received packets to a network server, if they can receive them. As a result, there is no hard limit on the number of end devices that can be supported by a single gateway and obviously depends on the load per device. Thus, if each device sends x packets per day, the limit per uplink channel is 90,000 × 24/(8*×), which, for example, equals to 90.000 devices that send two messages per day or 180.000 devices sending a message per day. Thus, there is no hard upper limit for supported devices per gateway per se, but rather the upper limit on devices in each case stems from the overall load, as also shown in [41], which has to be kept around 0.5 when normalized, due to the use of the Slotted ALOHA MAC that gives rises to many collisions.

## 6. Simulation Results

Using simulation, the proposed approach is compared against the static approach of [39,40] which employs the WuR-based Broadcast TDMA approach and Listen-Before-Talk (LBT) approach. In order to develop and evaluate the proposed protocol, a network simulator, using Java Programming Language has been implemented. LoRaWAN Class A has been modelled in the simulation analysis.

The following simulation environment is employed. Consider that *N* denotes the total number of end devices and *I* denotes the duration of a cycle. With the term “cycle”, it is defined whenever the automaton selects an action. For the simulation it was determined that L=0.1, $\alpha =\;10^{-4}$ and I=N∗M+ToA+WU, where *M* represents the slot’s size, (measured in ms), *ToA* stands for Time-on-Air (also measured in ms) and *WU* represents the time it takes to transmit a wake-up message. The simulation lasts 1000 cycles. Between the transmissions, a guard-time is added, which is equal to 6 ms. The guard-time guarantees that the window is large enough for the transmission and compensates for clock drift. In addition, the wake-up beacons last 17 ms. The cluster head is randomly selected and does not change, according to [39,40], which is the used architecture for increasing energy saving in end devices.

The initial probability distribution vector is formulated as $p\left(n\right)=\left\{\frac{1}{2},\frac{1}{2}\right\}$. After each cycle, it is updated based on Equation (2), using the feedback mechanism described in Section 5. Each node in the network generates a new packet at some time during each cycle. Event packets are generated by some nodes based on the traffic load. For example, in case traffic load is equal to 0.1, then 10% of the network nodes generate event packets. These nodes are the same during the whole simulation to present that an event is detected in a specific area of the network. All the other nodes generate regular packets. Regular packets are transmitted when TDMA is selected. The main goal is to transmit event packets as fast as possible to decrease their delay.

The three systems are compared considering three different LoRa radio settings (SETS) at low, medium and high data rate. These three configurations are summarized in Table 1 and are utilized according to [39,40] that describe the architecture the suggested paper was built on, while also describing the comparable MAC protocol. The Data Rate included in Table 1 is the same in each SET irrespective of the network load. In each experiment, the average delay of the system, the average number of collisions and the system throughput are measured as the number of end devices *N* and the traffic load increase. The selection rate of each MAC protocol is also reflected. Delay is defined as the time from the moment an event packet was generated at the end node until the moment it was successfully received by the gateway.

**Figure 5:**
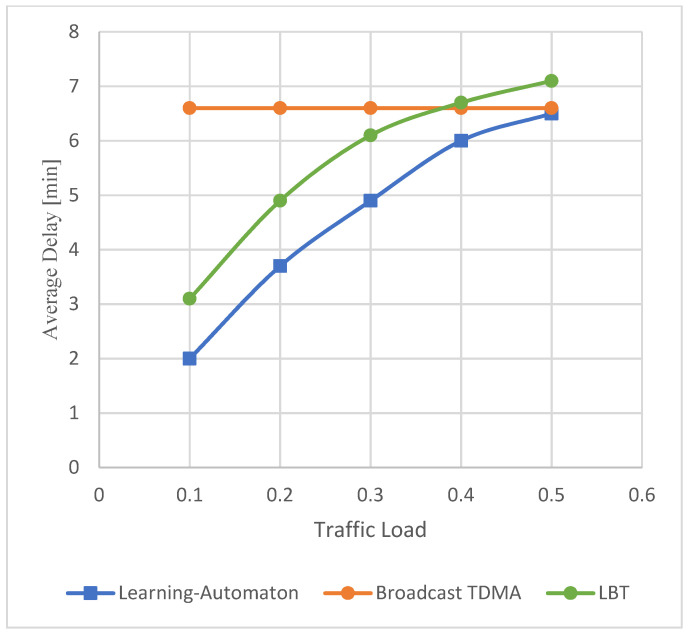
Average delay versus traffic load characteristics.

In Figure 5, event packet average delay, measured in minutes, versus traffic load is depicted, regardless of the simulated LoRa setting (SET 1, SET 2, SET 3). In this experiment, 2500, 17,500 and 40,000 end devices were used in total, corresponding to each SET, respectively. The traffic load is displayed on the x axis, while the average delay appears on the y axis. In this figure, it is recorded that Broadcast TDMA delay remains stable, as is also shown analytically in [39,40]. As previously stated, the average delay is defined as the average of the delay among all received event packets, with the delay for each event packet being equal to the time span between its generation at the end node and its successful reception at the gateway. Moreover, average delay includes retransmission time. Furthermore, it is observed that LBT average delay increases as the increased traffic load corresponds to more end devices attempting to transmit data resulting in an increased number of retransmissions.

From the perspective of Broadcast TDMA, the average delay metric is not affected by the nodes that did not generate any event packets. Based on the above equation, the behavior of Broadcast TDMA curve is justified. Regarding the proposed learning-automaton approach, it can be seen to perform significantly better than the Broadcast TDMA and LBT when the percentage of the network that needs to transmit an event packet is almost less than 50%. However, given that this model is proposed for events such as forest fire detection, a value of 50% is already an extreme scenario, meaning that it covers a large area. This is the reason why the comparable systems are simulated until the network traffic load reaches 50%. As a result, when the traffic is low, the learning automaton selects Slotted ALOHA more often, and thus the average delay is reduced compared to Broadcast TDMA, where the node must wait for its turn, in order to transmit, and LBT. It becomes clear that the suggested approach is more efficient in the context of delay.

Unlike Broadcast TDMA, the average delay of the learning-automata-based approach increases gradually for an increasing traffic load. This happens because in low traffic loads the automaton selects Slotted ALOHA for low delay. Consequently, due to more collisions, end devices are forced to use a backoff algorithm and wait for some random amount of time, in order to try to retransmit their event packets.

**Figure 6:**
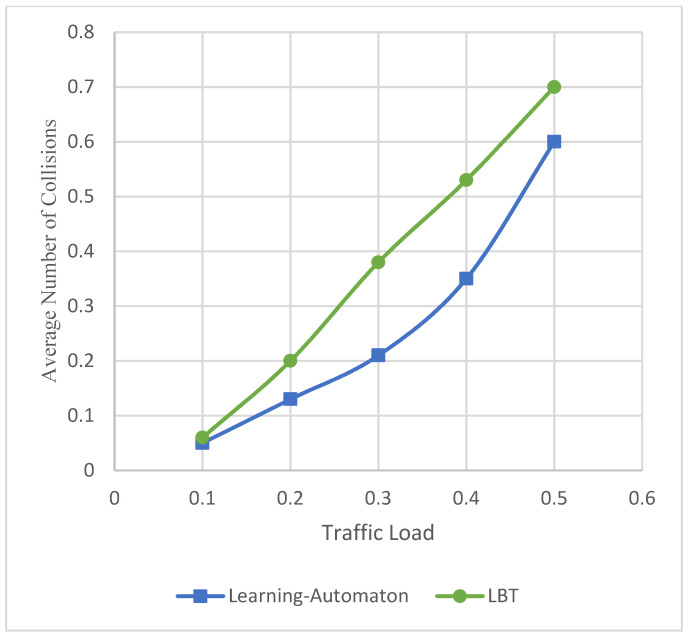
Average collisions versus traffic load.

Figure 6 represents the average number of collisions that refer to the proposed hybrid model and LBT, regardless of the simulated SET. It is shown that in both comparable systems, the average number of collisions gradually increases. This is happening because as the more end devices attempt to communicate, the more collisions occur. However, the learning automaton provides less collisions per packet comparing to the LBT method. This is happening because in the LBT approach, a collision may also occur between an uplink and a downlink packet.

**Figure 7:**
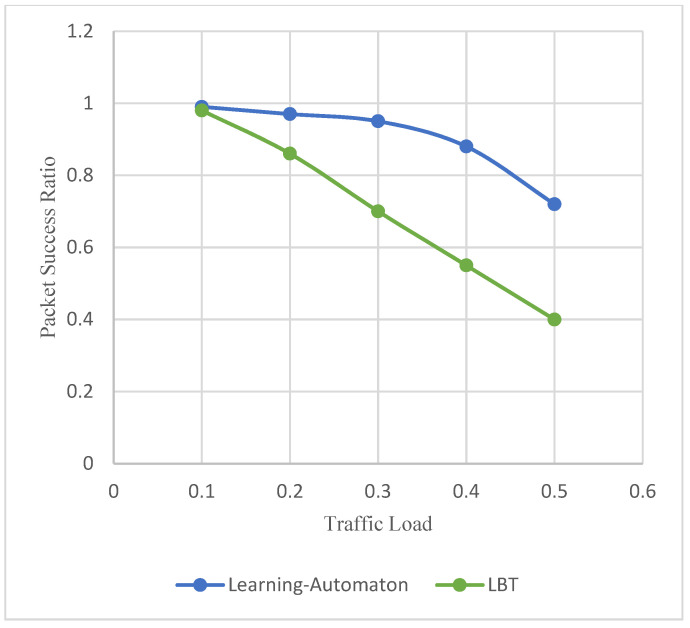
Packet success ratio versus traffic load.

Moreover, Figure 7 depicts the packet success ratio, as the traffic load increases, regardless of the simulated SET. The packet success ratio appears on the y axis, while the traffic load appears on the x axis. Packet success ratio is defined as the percentage of event packets that have been successfully transmitted. It is observed that the success ratio decreases in both approaches because as more end devices compete for the channel, more frequency collisions occur, and some packets are lost. Broadcast TDMA is collision-less by default, achieving the maximum success ratio. On the other hand, LBT performs in the worst possible way compared to the other two systems. Nevertheless, the learning automaton achieves a significantly high packet success ratio, which is particularly important, as no large amount of information is lost. Figure 6 and Figure 7 are part of the previous experiment, using 2500, 17,500 and 40,000 end devices in each SET, respectively. Furthermore, the maximum throughput achieved by the learning automaton is defined at 29%. However, Broadcast TDMA achieves 50% maximum throughput.

**Figure 8:**
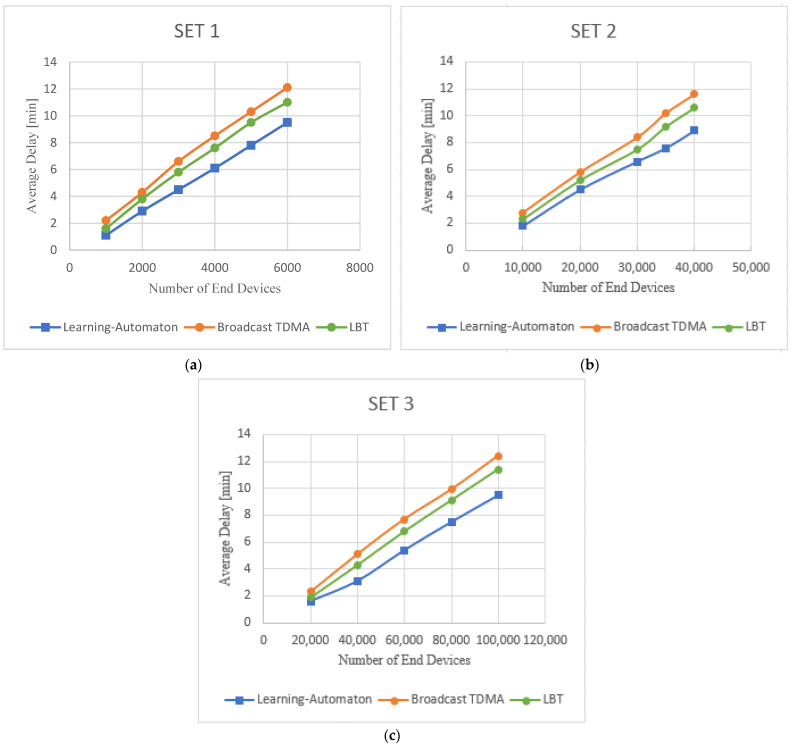
Average delay versus number of end devices for (**a**) SET1, (**b**) SET2 and (**c**) SET3.

In Figure 8a–c, the results from a new experiment are presented for the same three LoRa settings. More specifically, the average delay of the three comparable systems is recorded (y axis), while the number of end devices (x axis) increases. In this experiment, traffic load is constant and equal to 0.2. Average delay increases in all systems. This is an expected result, because as more end devices constitute the network, the more time it takes for all packets to be transmitted. Nonetheless, in the case of the proposed learning-automata-based mode, the average delay is much lower than the delay of the Broadcast TDMA and LBT methods, for a given number of end devices. Thus, for networks with many end devices, where low delay is required in the transmission of data, the proposed system responds more efficiently. This information is particularly important in applications such as fire detection in forest environments, where the network will consist of many end devices. However, the number of end devices has been selected in such a way that the network can no longer operate as it reaches unacceptable time limits for fire monitoring systems, which is estimated at about 10 min [10,11,12]. It is also clarified that in order to reach this time limit, the suggested approach is able to support more end devices, and as a result, it can be used for the coverage of larger geographical areas. It is also shown that the proposed learning automaton achieves an improvement ranging from 20% to 27% compared to Broadcast TDMA, in terms of delay. These percentages fluctuate between 15% and 25% when compared to LBT.

**Figure 9:**
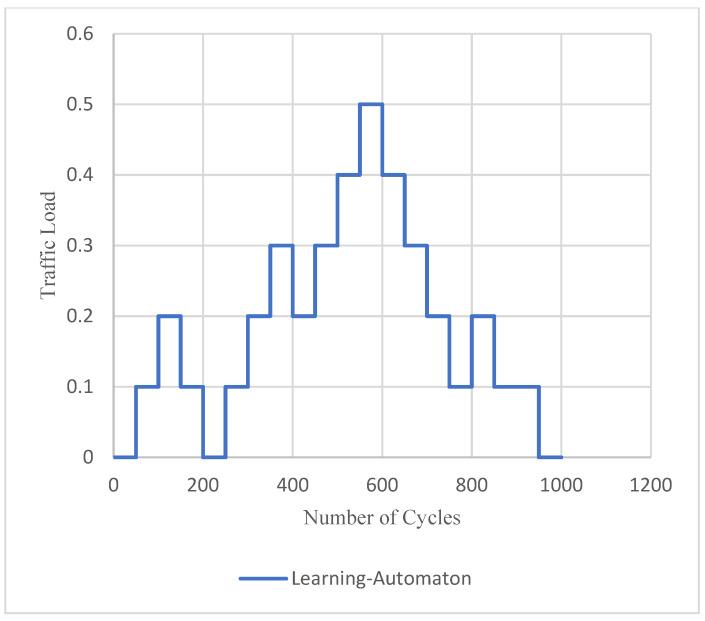
Event traffic load versus number of cycles.

**Figure 10:**
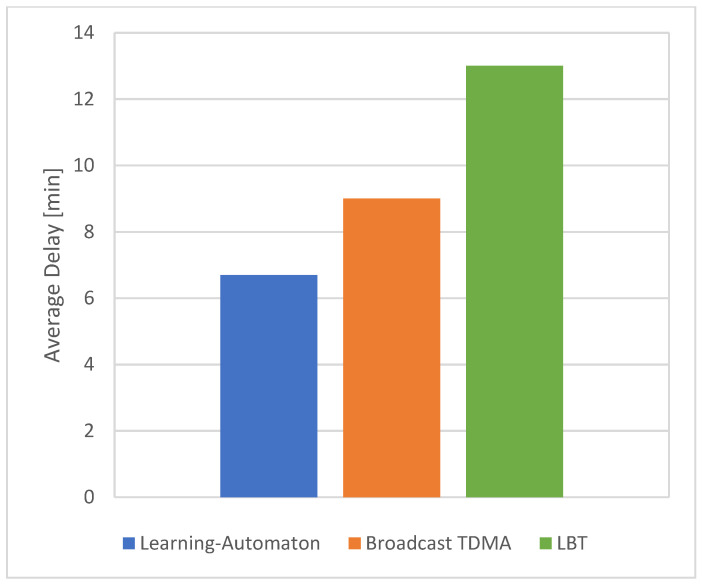
Average delay for variable traffic load for each SET.

The results of a new experiment for the three LoRa settings are presented in Figure 9 and Figure 10. The traffic load during the simulation is depicted in Figure 9, regardless of the simulated SET and the evaluated system. This dynamically adjusted event traffic load is simulated in each SET and for each method, among the proposed learning automaton, Broadcast TMDA and LBT. It is clarified that the traffic load is variable and changes dynamically to represent a real event detection scenario. Additionally, the three systems are compared in terms of average delay, which in turn is presented in Figure 10. Consequently, Figure 9 and Figure 10 illustrate results where the traffic load changes are produced by an increasement/decrement in event packet production from the network nodes. As a result, the number of end devices that produce urgent traffic is not stable during the simulation, and instead, it dynamically changes, according to Figure 9. All previous experiments correlated with static event traffic load. It is shown that the suggested learning-automata-based hybrid model provides significantly better performance. The main goal of this experiment is to evaluate the three comparable systems under the assumption of unknown and dynamic environment demands. Unknown environment demands implies that the learning-automata mechanism is not aware of future changes. Additionally, dynamic environment demands imply that the traffic load is dynamically changed, as shown in Figure 9. For this experiment, 2500, 17,500 and 40,000 end devices were used in total, corresponding to each SET, respectively (SET 1, SET 2, SET 3).

**Figure 11:**
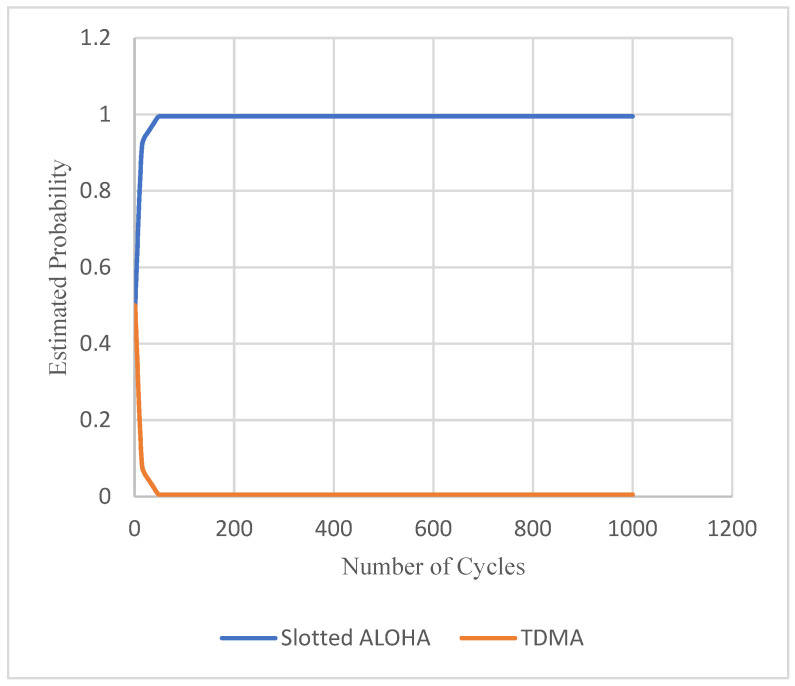
Convergence of automaton estimation of MAC protocol selection probabilities.

Figure 11 imprints the convergence of automaton estimation of MAC protocols’ selection probabilities, regardless of the simulated SET. The estimated selection probability of each MAC protocol toward the number of cycles is plotted. It appears that Slotted ALOHA is almost always chosen, because all simulations were performed under the assumption that there are always some event packets. In case there were long intervals without any event packet, then TDMA would sometimes be selected. Obviously, the speed of convergence also depends on the load of the event packets, as more event packets lead to faster convergence. For this experiment, the traffic load is stable and equals to 0.2. Thus, in such a situation, due to the transmission of event packets and the decreased contention due to their low load, the network feedback switches from TDMA to the use of Slotted ALOHA mechanism.

**Figure 12:**
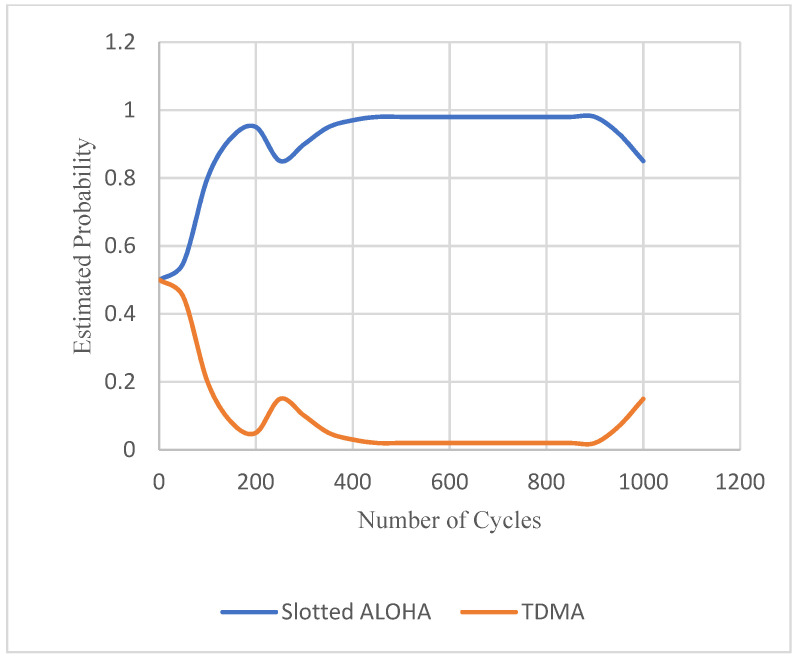
Convergence of automaton estimation of MAC protocol selection probabilities for variable traffic load.

In the case of evaluation of the variable traffic load scenario, shown in Figure 9, the convergence of automaton estimation of MAC protocols’ selection probabilities is imprinted in Figure 12. In this figure, it is observed that when the traffic load is non-zero, Slotted ALOHA is selected for the reason explained above. When the event load reaches zero, the learning automaton starts to select TDMA increasingly, until the event load rises to non-zero values again, as for example between cycles 200–250 and 950–1000. In cases where a zero value of event load would persist, for example after the 1000th cycle, the selection probability of TDMA would approach one, where that of Slotted ALOHA would approach zero.

## 7. Conclusions

To limit the damage caused by events such as forest fires and to control their start and spread via IoT-capable sensors, in this work, a learning-automata-based hybrid MAC model for LoRa networks is proposed. The automaton manages to lead to a decrease in the transmission delay of event packets, when a small part of the network produces urgent traffic, as would be the case of an event occurring over a large, monitored area. The proposed system has been compared with Broadcast TDMA and LBT. The simulation results clarify that the suggested approach is able to support more end devices, until the network reaches unacceptable time limits and can no longer operate, and as a result, a larger geographical area can be monitored. Furthermore, it provides an improvement up to 27% in terms of delay compared to Broadcast TDMA. In future work, a possible path could be the joint use of our approach with one such as [20], in a network that can switch to the best-suited MAC according to the network current state by our approach and also select the best operating parameters according to [20].

## Figures and Tables

**Table 1:** LoRa radio settings (SETs).

LoRa Radio Settings	SET 1	SET 2	SET 3
Spreading Factor	12	9	7
Coding Rate	4/6	4/5	4/5
Bandwidth (kHz)	500	500	500
Data Rate (kb/s)	0.976	7.03	21.87
Transmission Power (dBm)	10	10	10
Payload (B)	8	8	8
Preamble Length (symbol)	8	8	8
Carrier Frequency (MHz)	868	868	868
Time-on-Air (ms)	264	31	9

## Abbreviations

The following abbreviations are used in this manuscript:

IoT	Internet of Things
LoRaWAN	Long-Range Wide Area Network
MAC	Media Access Control
LPWAN	Low-Power Wide Area Network
WSN	Wireless Sensor Network
TDMA	Time Division Multiple Access
AODV	Ad hoc On-Demand Distance Vector
SMO	Sequential Minimal Optimization
LoRa	Long Range
LA	Learning Automaton
CSS	Chirp-Spread-Spectrum
T_s_	Symbol Time
SF	Spreading Factor
BW	Bandwidth
CR	Coding Rate
FEC	Forward Error Correction
R_s_	Symbol Rate
ToA	Time-on-Air
CRC	Cyclic Redundancy Check
MAC	Medium Access Control
WuR	Wake-up Radios
CH	Cluster Head
LBT	Listen-Before-Talk
ACK	Acknowledgement
